# Hybrid and herd immunity 6 months after SARS-CoV-2 exposure among individuals from a community treatment program

**DOI:** 10.1038/s41598-023-28101-5

**Published:** 2023-01-14

**Authors:** Parawee Chevaisrakul, Putthapoom Lumjiaktase, Pongtorn Kietdumrongwong, Ittiporn Chuatrisorn, Pongsan Chatsangjaroen, Nittaya Phanuphak

**Affiliations:** 1 Allergy Immunology and Rheumatology Division, Internal Medicine Department, Faculty of Medicine, Ramathibodi Hospital, Mahidol University, Bangkok, Thailand; 2 Pathology Department, Faculty of Medicine, Ramathibodi Hospital, Mahidol University, Bangkok, Thailand; 3 BDMS Health Research Center, Bangkok Dusit Medical Service, Bangkok, Thailand; 4 National Healthcare Systems Company Limited, Bangkok, Thailand; 5 Institute of HIV Research and Innovation, Bangkok, Thailand

**Keywords:** Immunology, Molecular medicine

## Abstract

The death rate from severe acute respiratory syndrome coronavirus 2 (SARS-CoV-2) infections in 2022 was lower than the death rate in 2021, when the infection rate increased. Hybrid immunity provided by a combination of vaccination and infection, including asymptomatic infection, may confer effective protection against death. We explored the combined effect of asymptomatic infection and hybrid immunity by studying T-cell and antibody responses against SARS-CoV-2 among individuals treated in home health care services 6 months after SARS-CoV-2 exposure. Asymptomatic SARS-CoV-2 infection was demonstrated in 24.4% of close contacts. The levels of immunity were not different between patients and close contacts. Anti-RBD IgG against SARS-CoV-2 increased in a dose-dependent manner with the number of vaccine doses. Interestingly, the T-cell response decreased soon after a booster dose of vaccine. Asymptomatic SARS-CoV-2 infection could not enhance immunity against SARS-CoV-2 among vaccinated close contacts. Full vaccination was crucial to provide hybrid immunity. However, when designing vaccine strategies, T-cell exhaustion after multiple vaccinations should be considered.

## Introduction

Since early 2020, severe acute respiratory syndrome coronavirus 2 (SARS-CoV-2) has spread worldwide, becoming a pandemic. By mid-August 2021, Thailand faced the Delta variant of SARS-CoV-2, with the infection rate reaching over 20,000 cases per day and approximately 300 daily confirmed deaths^1^. Multiple measures were implemented to reduce the infection rate and mortality, which included a nationwide vaccination program that prioritized populations at high risk of developing severe illness. A decline in the infection rate was observed together with greater adoption of vaccines, especially mRNA vaccines.

The Centers for Disease Control (CDC) reported that 66% of Americans were fully vaccinated and had a much lower mortality rate than unvaccinated individuals (0.1 vs. 0.76 per 100,000 people); however, mRNA vaccines have been used as the primary vaccine option in the United States. In contrast, 91% of Chile’s population was fully vaccinated but mostly with inactivated vaccines. The difference in the Chilean mortality rate was less significant between the fully vaccinated and unvaccinated groups (0.41 vs. 0.8 per 100,000 people) when compared to the US^2^.

In the last trimester of 2022, more than 75% of the population (70 million people) in Thailand had been fully vaccinated, mostly with viral vector and mRNA vaccines. The SARS-CoV-2 Omicron variant surged in 2022, and to date, 4 million people in Thailand have been confirmed to be infected (5% of the population). Again, new cases of SARS-CoV-2 infection or recurrent cases of infection increased to 50,000 per day. However, the death rate was lower (120 per day) compared to the death rate during the peak of the Delta variant (300 per day)^1^.

Many scientists expected herd immunity to develop after most of the population was immunized, either through vaccine-generated immunity or natural immunity against SARS-CoV-2 infection. Nonetheless, the herd immunity threshold was unlikely to be met because of vaccine hesitancy and the emergence of new variants. Long-term prospects for the pandemic may be the influence of SARS-CoV-2 as an endemic disease and contemplating a new normal that does not include herd immunity. Interestingly, evidence from a United Kingdom study^3^ revealed that “hybrid immunity” (defined as immunity against SARS-CoV-2 generated by heterogeneous prime-boost vaccines in combination with SARS-CoV-2 infection) was long-lasting and conferred highly effective protection against symptomatic disease for at least 6–8 months after vaccination before the arrival of Omicron.

We observed a lower infection rate among close contacts of SARS-CoV-2 patients who received favipiravir in a home-based health care setting during the Delta outbreak in Bangkok. During the outbreak, more than 30,000 cases were treated at home, with a lack of social distancing due to space constraints and the number of infected cases within the same household. We hypothesized that the lower death rate may have been due to herd immunity and hybrid immunity in addition to the lower virulence of Omicron. Moreover, evidence revealed that (memory) T-cell immunity against SARS-CoV-2 developed in close contacts, who were considered asymptomatic cases^4^. These asymptomatic infections may contribute to some degree of herd immunity and hybrid immunity in society. Therefore, we studied T-cell immunity and antibody responses against SARS-CoV-2 among patients and close contacts who were registered in the Bangkok home health care service 6 months after SARS-CoV-2 exposure.

## Materials and methods

### Subjects

This observational study enrolled 79 participants from 15 families who were randomly invited from different Bangkok metropolitan regions. In each family, there had to be at least one SARS-CoV-2-infected patient registered in a database of the Bangkok home health care service between 1 and 31 August 2021. In addition, there had to be at least 1 asymptomatic close contact with negative antigen living in the same accommodation following the national health policy regulation. Thirty-four cases were individuals who had recovered from SARS-CoV-2 at least 4 weeks before enrollment, while 45 cases were close contacts. The calculated sample size was 90 cases based on the prevalence of asymptomatic SARS-CoV-2 among close contacts reported in the Chinese population^4^, with 80% power to detect asymptomatic infection. Peripheral blood (15 ml) was collected after obtaining informed consent from participants. This study was approved by the ethics committee of Ramathibodi Hospital, Mahidol University (MURA2021/923) and Bangkok Hospital (BHQ-IRB 2021-11-34) according to the Declaration of Helsinki, the Belmont Report, CIOM Guidelines and the International Conference on Harmonization in Good Clinical Practice (ICH-GCP).

### Materials and reagents

A FACSLyric™ (Becton Dickinson, USA) flow cytometer was employed for cytokine detection, and Beckman Coulter’s FC 500 series (Beckman Coulter, USA) was employed for CBC determination. The Alinity and ARCHITECT Systems were used for IgG levels against SARS-CoV-2, and the EUROIMMUN Analyzer I was used for the ELISA reader. The incubator, centrifuge, vortex mixer and automatic cell counter were from Thermo Fisher Scientific, Inc. (USA). Phosphate-buffered saline (PBS) with a pH of 7.4 was purchased from Sigma‒Aldrich (USA), heparin and EDTA tubes were purchased from Becton Dickinson (USA), and RPMI-1640 medium was purchased from Life Technologies (USA).

### The enzyme-linked immunosorbent spot (ELISpot) assay and T-SPOT

Fresh heparinized whole blood samples (10 mL) from volunteers were isolated for peripheral blood mononuclear cells (PBMCs) using SepMate PBMC isolation tubes (STEMCELL Technologies Inc., Canada). The heparinized blood was diluted with RPMI medium (1:1) in SepMate PBMC isolation tubes and centrifuged at 1700*g* at 20 °C for 20 min. The separate PBMCs were washed twice with PBS and recentrifuged at 500×*g* for 5 min at 4 °C. PBMCs (2.5 × 10^5^ cells) were added to 96-well plates precoated with an anti-IFN-g antibody of the T-SPOT^®^ COVID test (Oxford Immunotec, Ltd., UK). The plate stimulated each sample composed of four wells with two antigens against the spike (S) protein and nucleocapsid (N) protein, membrane glycoprotein (M) and ORF1ab region of RNA-dependent RNA (O) of the SARS-CoV-2 Alpha variant; phytohemagglutinin (PHA) and medium alone were used as the positive and negative controls, respectively. Plates were maintained overnight at 37 °C in a 5% CO_2_ humidified atmosphere, washed with phosphate-buffered saline and developed using an anti-IFN-g antibody conjugate and substrate to detect the presence of secreted IFN-g. Spot-forming cells (SFCs) were counted with an automated ELISpot reader (CTL Analyzers, Cleveland, OH, USA). Less than 10 SFCs per 250,000 cells was represented as a normal background according to the manufacturer’s recommendations that was comparable to the lowest quartile of SFCs in SARS-CoV-2 patients in this study.

### IgG level of SARS-CoV-2 in the receptor-binding domain (RBD)

Human EDTA plasma samples were measured to quantitatively determine IgG antibodies against the spike receptor-binding domain (RBD) to SARS-CoV-2, which used the SARS-CoV-2 IgG II Quant assay on the Alinity and ARCHITECT I Systems. The chemiluminescent reaction was calculated as a relative light unit (RLU) and expressed as a calculated index (S/C). The SARS-CoV-2 IgG II Quant assay cutoff was 50 AU/mL, and those greater than 50 AU/mL were interpreted as positive.

### ELISA to detect SARS-CoV-2-specific neutralizing antibodies

Human EDTA plasma samples were diluted 1:5 in sample buffer, which was performed according to the manufacturer’s instructions for the Euroimmun SARS-CoV-2 NeutraLISA (Euroimmun AG, Lübeck, Germany). In brief, 100 μL of the diluted sample, control, or blank was added per well and incubated at 37 °C for 1 h. The automatic machine washed the plate 3 times with wash buffer; then, 100 μL of enzyme conjugate was added and incubated at RT for 30 min. After the washing cycle, 100 μL of substrate solution was added, and the plate was incubated at RT for 15 min. Finally, 100 μL of stop solution was added per well, and the absorption at 450 nm was measured using a EUROIMMUN Analyzer I. Samples were analyzed in a single replicate. The percent inhibition (%IH) was calculated as follows: 100% − [(extinction of sample × 100%)/extinction of blank]. The Euroimmun recommends interpreting results as follows: %IH < 20: negative; %IH > 20 to < 35: borderline; and %IH > 35: positive.

### Statistical analysis

Descriptive results are presented as the medians (interquartile ranges) and percentages ± standard deviations. Corresponding inferential comparisons were performed using the t-test or Mann‒Whitney U test, and the correlation analysis was calculated using GraphPad Prism version 9.4.0 for Window, GraphPad Software, San Diego, California USA, www.graphpad.com.

## Results

### Asymptomatic SARS-CoV-2 infection among close contacts

**Figure 1:**
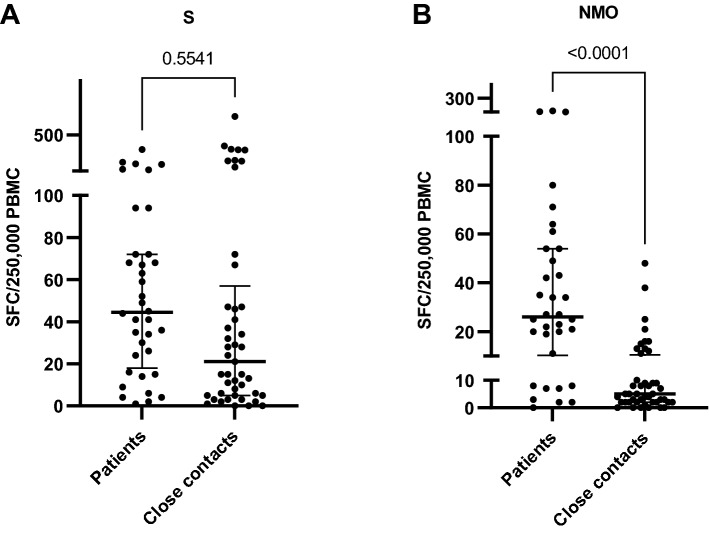
T-cell response against the spike (S) protein (**A**) and nucleocapsid (N) protein, membrane glycoprotein (M) and ORF1ab region of RNA-dependent RNA (O) of SARS-CoV-2 (**B**) evaluated by ELISPOT (P < 0.0001, t = 5.720, df = 77). *SFCs* spot-forming cells, *PBMCs* peripheral blood mononuclear cells.

During the SARS-CoV-2 exposure period, patients and close contacts in each family of this study were living in the same accommodation. There were 15 families comprising 11 individuals (median, range 3–30) per family living in a space of 200 m^2^ (median, range 30–400 m^2^). Most of the participants were female (58%), younger than 60 years (91%) and had a body mass index less than 30 (81%). Comorbidities, defined as any risk of severe SARS-CoV-2 infection, were detected in one-fifth of the participants (26.5% in patients and 15.5% in close contacts) (Table 1). Our data showed that the T-cell response to the S antigen in close contacts was not different from that in recovered SARS-CoV-2 patients (Fig. 1). Since 75% of participants were fully vaccinated, the T-cell response to the S antigen in this study population may have been either the result of previous infection or vaccination. Therefore, we analyzed the T-cell response against NMO antigens and found a positive T-cell response (at a cutoff of 10 SFCs/250,000 cells) in 11 of 45 cases (24.4%) of close contacts, which was considered evidence of previous infection (Fig. 1). However, a history of vaccination with a whole virus molecule (inactivated vaccine) may have been a contradicting factor. One-fourth of the participants were fully vaccinated with an inactivated vaccine 3.5 months before enrollment. To explore this issue, we analyzed the history of vaccination among 11 close contacts and found only 2 cases that received an inactivated vaccine. Therefore, the T-cell responses to the NMO antigen in these cases were most likely due to asymptomatic SARS-CoV-2 infections.

**Table 1:** Demographic data.

Demographic data	Total number (%)	Patients (%)	Close contacts (%)
**SARS-CoV-2 viral exposure**	79	34	45
**Sex**			
Female	46 (58.2)	23 (67.6)	23 (51.1)
**Age**			
18–60 years	72 (91.2)	30 (88.2)	42 (93.3)
> 60 years	7 (8.8)	4 (11.8)	3 (6.7)
**BMI**			
< 25	44 (55.7)	14 (41.2)	30 (66.6)
25–30	20 (25.3)	13 (38.2)	7 (15.5)
> 30	15 (19)	7 (20.6)	8 (17.9)
**Risk factors for severe SARS-CoV-2 infection**	15 (18.9)	9 (26.5)	7 (15.5)
Coronary artery disease, CAD	3	1	2
Diabetes mellitus, DM	7	6	1
Chronic lung disease	1	0	1
Chronic kidney disease	1	1	0
Cancer	1	1	0
DM with CAD	1	0	1
DM with asthma	1	0	1
**Number of vaccine doses**			
0	13 (16.4)	4 (11.8)	9 (20)
1	7 (15.5)	4 (11.8)	3 (6.7)
2	35 (44.3)	13 (38.2)	22 (48.9)
3	18 (22.8)	9 (26.4)	9 (20)
4	6 (1)	4 (11.8)	2 (4.4)
**Type of vaccine**			
1st dose	66	30	36
Inactivated vaccine	23 (34.8)	9 (30)	14 (38.9)
Viral vector vaccine	39 (59.1)	18 (60)	21 (58.3)
mRNA vaccine	4 (6.1)	3 (10)	1 (2.8)
2nd dose	59	34	25
Inactivated vaccine	19 (32.2)	16 (47)	3 (12)
Viral vector vaccine	32 (54.2)	12 (35.3)	20 (80)
mRNA vaccine	8 (13.6)	6 (17.7)	2 (8)
3rd dose	24	9	15
Viral vector vaccine	7 (29.2)	4 (44.5)	3 (20)
mRNA vaccine	17 (70.8)	5 (55.5)	12 (80)
4th dose	6	4	2
Viral vector vaccine	1 (16.6)	1 (25)	0 (0)
mRNA vaccine	5 (83.4)	3 (75)	2 (100)
**Diagnostic test for SARS-CoV-2 infection**			
Rapid antigen test		33	
RT–PCR		13	
**Treatment**			
Favipiravir		28	
Remdesivir		2	
Corticosteroids		4	
None		5	
Referred to hospital		8	
**Accommodation**			
Living area			
30–60 m^2^	13 (16.5)		
80–200 m^2^	30 (38)		
200–400 m^2^	36 (45.5)		
**Number of family members**			
3–5	32 (40.5)		
6–15	17 (21.5)		
16–30	30 (38)

### Hybrid immunity and herd immunity against SARS-CoV-2 in recovered patients and close contacts

In this study population, the number of vaccine doses was stratified by a group of patients (with previous SARS-CoV-2 infection) and a group of close contacts (no history of previous SARS-CoV-2 infection), as shown in Table 1. There were 4 unvaccinated patients and 9 unvaccinated close contacts. Four individuals received one vaccine dose among 7 patients and 9 among 13 close contacts, 13 received 2 vaccine doses among 35 patients and 22 among 35 close contacts, 9 received 3 vaccine doses among 18 patients and 9 among 18 close contacts, and 4 received 4 vaccine doses among 6 patients and 2 among 6 close contacts.

**Figure 2:**
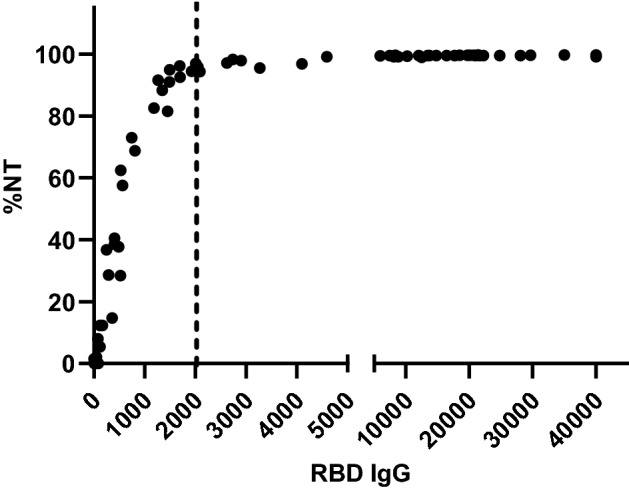
The antibody response to RBD IgG demonstrates the correlation between the antibody levels and neutralization capacity against the Alpha variant of SARS-CoV-2 (R = 0.5571, P < 0.0001).

**Figure 3:**
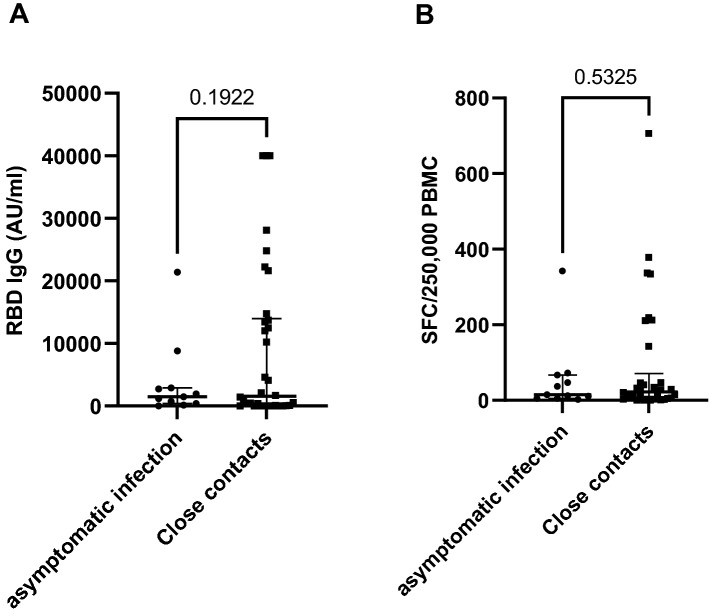
Immune response against SARS-CoV-2 viral antigens between close contacts with and without asymptomatic infection, antibody response (RBD IgG) P = 0.1922 t = 1.325, df = 43 (**A**), T-cell response against the spike protein P = 0.5325, t = 0.6293, df = 43 (**B**). *RBD IgG* SARS-CoV-2 receptor-binding domain immunoglobulin G, AU/ml = arbitrary units per milliliter.

The percentage of neutralizing antibody (%NT) against the Alpha variant of SARS-CoV-2 correlated with the levels of RBD IgG (Fig. 2). At 6 months after SARS-CoV-2 exposure, unvaccinated patients had very low levels of RBD IgG, comparable to those of close contacts. In addition, the T-cell and antibody responses against SARS-CoV-2 in close contacts with asymptomatic infection (with a positive T-cell response to NMO antigens) were not different from other cases of close contacts (Fig. 3).

**Figure 4:**
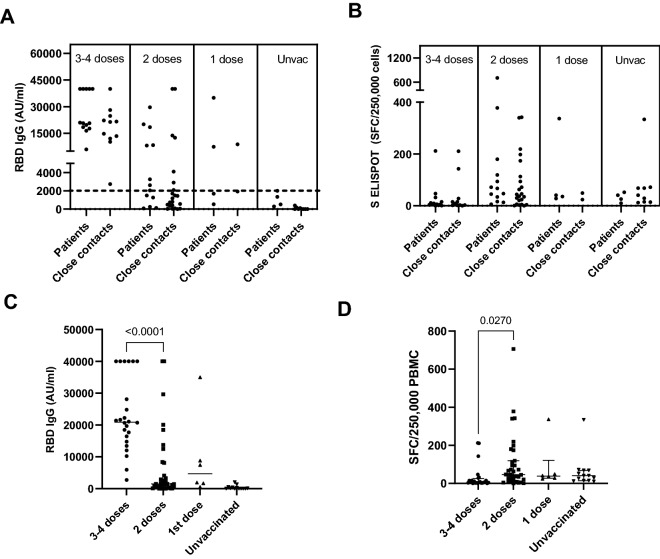
The antibody response (**A**) and T-cell response (**B**) against SARS-CoV-2 in patients (previous infection) and close contacts (noninfection) were stratified by the number of vaccine doses. There were no significant differences in the antibody response or T-cell response between patients and close contacts stratified by the number of vaccine doses. The antibody response against SARS-CoV-2 in patients and close contacts increased in a dose-dependent manner according to the number of vaccine doses. There was a significant difference in RBD IgG levels between those who received 2 doses vs. 3–4 doses (P < 0.0001, t = 5.682, df = 57) (**C**). The T-cell response against the spike protein (S ELISPOT) significantly decreased after the booster dose of the vaccine, P = 0.027, t = 1.961, df = 46 (**D**).

T-cell and antibody responses against SARS-CoV-2 demonstrating hybrid immunity and herd immunity are depicted in Fig. 4. Hybrid immunity was identified among vaccinated patients with previous SARS-CoV-2 infection, while herd immunity was identified among unvaccinated patients with previous SARS-CoV-2 infection and vaccinated close contacts without a history of SARS-CoV-2 infection. In addition, we observed 9 close contacts who were unvaccinated. In theory, these patients could not develop immunity against SARS-CoV-2 during the study period. However, some of them demonstrated a T-cell response against the S antigen, as shown in Fig. 4B. Thus, it is possible that they may have developed herd immunity from asymptomatic SARS-CoV-2 infections.

After vaccination, increments of RBD IgG levels were observed in a dose-dependent manner according to the number of vaccine doses among both patients and close contacts. However, the T-cell response did not increase in the same pattern as RBD IgG. There was a significant decrease in T-cell responses against the S antigen in participants who received three and four doses of the vaccine.

**Figure 5:**
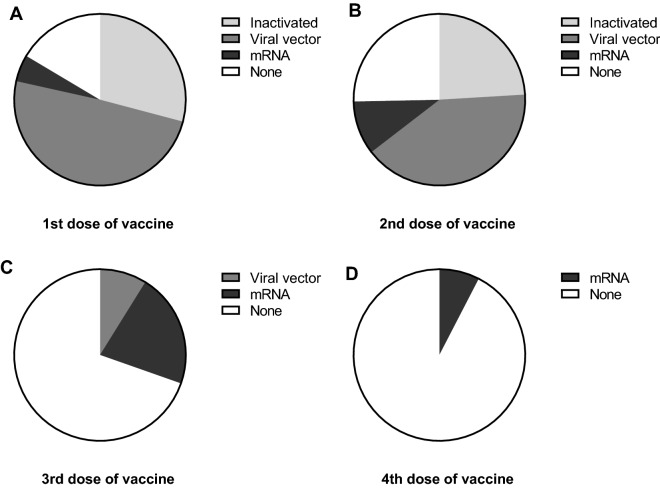
Types of vaccines against SARS-CoV-2 for participants are depicted in a pie chart to demonstrate the proportion of people who received the 1st dose (**A**), 2nd dose (**B**), and 3rd dose (**C**) to the 4th dose of vaccine (**D**) and inactivated vaccine, viral vector vaccine, and mRNA vaccine; None = unvaccinated individuals.

To explore vaccine type as a confounding factor, we evaluated the types of vaccines in the study population and found that 75% of participants were fully vaccinated with an inactivated vaccine or viral vector vaccine (3.5 months before enrollment), while less than one-third of participants received the third or fourth booster dose of an mRNA vaccine (1 month before enrollment) (Fig. 5). The numbers and types of vaccines among patients and close contacts are also described in the demographic data (Table 1).

## Discussion

“Herd immunity” is developed through either vaccine-generated immunity or natural immunity against SARS-CoV-2 infection. “Hybrid immunity" (defined as immunity against SARS-CoV-2 generated by heterogeneous prime-boost vaccines in combination with SARS-CoV-2 infection) may confer highly effective protection against symptomatic disease after vaccination^3^. In addition, evidence has revealed asymptomatic SARS-CoV-2 infection in close contacts defined by the T-cell response against SARS-CoV-2^4^. Asymptomatic infections may contribute to some degree of hybrid immunity in society.

Six months after SARS-CoV-2 exposure, we observed a T-cell response against NMO antigens (interferon release assay)^5^ in 11 of 45 close contacts. The rate of asymptomatic SARS-CoV-2 infection in the study population was 24.4%, similarly to a previous study that reported 16–26% of asymptomatic SARS-CoV-2 infection in close contacts explored by (ex vivo) CD4 and CD8 memory T-cell responses^4^. Unfortunately, we did not observe a different immune response against SARS-CoV-2 generated after full vaccination in previously SARS-CoV-2-infected patients compared to close contacts, as shown by antibody and T-cell responses against the Alpha variant of SARS-CoV-2. In addition, we revealed similar immunity against SARS-CoV-2 between close contacts with and without asymptomatic infection (defined by T-cell response against NMO antigens). These results revealed that immunity against SARS-CoV-2 among participants was mainly stimulated by vaccination among both patients and close contacts. Nonetheless, we did not have observational data on the reinfection rate, and there were no participants who were reinfected with SARS-CoV-2 during the study period^3^.

In this study population, most of the participants received a heterogeneous prime-boost vaccine comprising an inactivated vaccine, viral vector vaccine and mRNA vaccine. A previous report revealed the high efficacy of a heterogeneous prime-boost vaccine in terms of neutralizing antibodies and T-cell responses^6^. However, we studied the neutralizing capacity of RBD IgG against the Alpha variant of SARS-CoV-2. Recently, evidence revealed a reduced neutralizing capacity of RBD IgG against mutant variants of SARS-CoV-2 compared to wild-type SARS-CoV-2 for different vaccination strategies, with or without previous infection^7,8^.

T-cell dysfunction occurs in the setting of prolonged antigen stimulation across chronic infection, cancer and autoimmunity. However, the clinical outcome may differ in each context. CD8 + T cells undergo a progressive loss of cytokine production and cytotoxicity, a state termed “T-cell exhaustion”^9^. After three and four doses of a vaccine, we observed a lower T-cell response against the spike protein, in contrast to higher RBD IgG levels. This contradicts data from a previous study that revealed correlations between the T-cell response to spike and nucleoprotein/membrane proteins and peak antibody levels^5^. Reduced T-cell response against SARS-CoV-2 (T-cell exhaustion) was observed 1 month after three and four vaccine doses. These data are contrary to those in a previous report that demonstrated that the CD4 + T-cell response, which was typically detectable by Day 8 after priming, peaked soon after the booster dose of the vaccine and then fell to prebooster levels after 4 months^10^. Nonetheless, after a short period of T-cell exhaustion, as observed in this study, the T-cell response could recover 3 months after a booster dose of vaccine, as shown in a report from Zuo et al. demonstrating antibody and T-cell responses against the SARS-CoV-2 Omicron variant after heterogeneous immunization with an inactivated vaccine followed by an mRNA booster^11^. Recent evidence from Reinscheid et al. also revealed that the spike-specific CD8 + T memory stem cell pool is not affected by the third dose of a vaccine. Moreover, the booster dose of a vaccine and breakthrough infections with Delta or Omicron variants of SARS-CoV-2 rapidly reactivated CD8 + T memory cells 3 months after the previous vaccination dose^12^.

Individuals may exhibit varying cellular and humoral immune responses during primary viral infection, with some patients displaying imbalanced viral-specific B-cell and T-cell immunity, especially individuals who suffer severe and long-lasting symptoms^13^. Since most of our study population received the mRNA vaccine as a booster dose, it is also possible that the mRNA vaccine hampers T-cell function after vaccination^14,15^. Nonetheless, this result raised concerns about T-cell exhaustion after multiple doses of a vaccine, especially soon after a short interval of revaccination (1–3 months). This observation might explain the evidence of herpes zoster reactivation after receiving the booster dose of a vaccine with transient lymphopenia^16^. Therefore, multiple vaccine doses should be used with special considerations in the case of immunocompromised hosts with poor T-cell response and closed monitoring for infection, especially early after a booster dose of vaccine. Recent evidence has addressed the crucial role of memory B cells in producing identical antibodies upon reinfection with the same virus and encoding a library of antibody mutations. Nonetheless, T cells are needed for the generation of diverse memory B cells^17^. Moreover, in the current situation with reduced neutralizing antibodies against the Omicron variant^9^, a T-cell-based vaccine is needed^10,18^.

Even with the limitations in this study, such as the small number of participants, long duration after SARS-CoV-2 exposure (6 months) and immune response confounded by vaccination, this study revealed the antibody and T-cell responses in symptomatic and asymptomatic SARS-CoV-2 infections, defined by individual immune responses in the community that may result in long-term protection as hybrid immunity. Designing vaccine strategies for the next pandemic is needed.

## Data Availability

The datasets used and/or analyzed during the current study are available from the corresponding author upon reasonable request.

## References

[1] https://ourworldindata.org/explorers/coronavirus-data-explorer.

[2] https://ourworldindata.org/covid-deaths-by-vaccination.

[3] Hall V, for the SIREN Study Group (2022). Protection against SARS-CoV-2 after Covid-19 vaccination and previous infection. NEJM.

[4] Wang Z (2021). Exposure to SARS-CoV-2 generates T-cell memory in the absence of a detectable viral infection. Nat. Commun..

[5] Zuo J (2021). Robust SARS-CoV-2-specific T cell immunity is maintained at 6 months following primary infection. Nat. Immunol..

[6] Atmar RL (2022). Homologous and heterologous Covid-19 booster vaccinations. N. Engl. J. Med..

[7] Cheng SMS (2022). Neutralizing antibodies against the SARS-CoV-2 Omicron variant BA.1 following homologous and heterologous CoronaVac or BNT162b2 vaccination. Nat. Med..

[8] Mattoo SS, Myoung J (2021). A promising vaccination strategy against COVID-19 on the horizon: Heterologous immunization. J. Microbiol. Biotechnol..

[9] Collier JL, Weiss SA, Pauken KE, Sen DR, Sharpe AH (2021). Not-so-opposite ends of the spectrum: CD8^+^ T cell dysfunction across chronic infection, cancer and autoimmunity. Nat. Immunol..

[10] Skelly DT (2021). Two doses of SARS-CoV-2 vaccination induce robust immune responses to emerging SARS-CoV-2 variants of concern. Nat. Commun..

[11] Zuo F (2022). Heterologous immunization with inactivated vaccine followed by mRNA-booster elicits strong immunity against SARS-CoV-2 Omicron variant. Nat. Commun..

[12] Reinscheid M (2022). COVID-19 mRNA booster vaccine induces transient CD8+ T effector cell responses while conserving the memory pool for subsequent reactivation. Nat. Commun..

[13] Moss P (2022). The T cell immune response against SARS-CoV-2. Nat. Immunol..

[14] Stuart ASV (2022). Immunogenicity, safety, and reactogenicity of heterologous COVID-19 primary vaccination incorporating mRNA, viral-vector, and protein-adjuvant vaccines in the UK (Com-COV2): A single-blind, randomised, phase 2, non-inferiority trial. Lancet.

[15] Pozzetto B (2021). Immunogenicity and efficacy of heterologous ChAdOx1-BNT162b2 vaccination. Nature.

[16] van Dam CS, Lede I, Schaar J, Al-Dulaimy M, Rosken R, Smits M (2021). Herpes zoster after COVID vaccination. Int. J. Infect. Dis..

[17] Crotty S (2021). Hybrid immunity, COVID-19 vaccine responses provide insights into how the immune system perceives threats. Science.

[18] Vardhana S, Baldo L, Moriceii WG, Wherry EJ (2022). Understanding T cell response to COVID-19 is essential for informing public health strategies. Sci. Immunol..

